# Case of Infantile Epilepsy

**Published:** 1856-12

**Authors:** Abraham Livezey

**Affiliations:** Lumberville, Penn.


Communicated for the Boston Medical and Surgical Journal.

### *Case of Infantile Epilepsy.* By ABRAHAM LIVEZEY, A. M., M. D., Lumberville, Penn.

Mrs. M. was confined with her third (male) child—the previous one being 10 years old—in June last; it was of average size, and, to all external appearances, as healthful looking as a majority of infants met with.

At my subsequent visit, 48 hours after delivery, the mother remarked that she thought the "baby was not quite right somehow." Upon inquiry, I learned that he had taken the breast, in fact had nursed well, for the milk was in abundance, and the meconium had passed freely. I therefore concluded that the mother's fears were groundless, unless the "something wrong" might have have been produced by overfeeding, and advised accordingly.

The next day, however, I was called in consequence of repeated convulsions having ensued. The infant still nursed well at stated intervals of three or four hours, slept well—would pass from a fit into a sleep, and from a tranquil sleep into a fit, apparently without pain. Meconium-like matter still passing freely; and under the presumption that this in excess might be the irritating cause of convulsions, I ordered twenty drops of ol. ricini, to be repeated if necessary, with much prudence exercised in reference to nursing. On the next day I was informed that though the evacuations were modified, yet the convulsions continued unabated. Upon a close examination, I could only detect a flatulent condition of the alimentary canal to prescribe for, and consequently ordered milk of assafœtida with friction externally. Still no alteration at the end of 48 hours, when finding some symptoms of acidity with biliary derangement, I ordered a small portion of hyd. c. creta, to be followed by twenty drops of oil, and by small portions of a mixture of soda, camphor, ipecac and opium every three or four hours. These were continued for two days without any alteration; at which time, thinking that "intestinal spasm" might be the cause, oil was again administered to remove all sources of irritation, followed by McMunn's elixir of opium as the mildest and safest anodyne, in small repeated doses, for several days—to be laid aside, if unsuccessful, for small doses of oxyde of zinc, advised by Dr. Rice, resident physician of the Western Infirmary, Philadelphia, which he had successfully employed in cases of infantile epilepsy, which from all the symptoms of the case, he was satisfied was of this nature. The zinc failing, I resolved to make a more decided impression upon the nervous system, and accordingly left a score of powders, containing a minute portion of copper, iron and quinine, one to be given every six hours. The convulsions began to abate in less than 24 hours, and in a few days had almost wholly disappeared. In ten days the infant was perfectly well.

